# The Analgesic and Antineuroinflammatory Effect of Baicalein in Cancer-Induced Bone Pain

**DOI:** 10.1155/2015/973524

**Published:** 2015-11-15

**Authors:** Shan Hu, Yu Chen, Zhi-Fu Wang, Qi-Liang Mao-Ying, Wen-Li Mi, Jian-Wei Jiang, Gen-Cheng Wu, Yan-Qing Wang

**Affiliations:** ^1^Department of Integrative Medicine and Neurobiology, State Key Laboratory of Medical Neurobiology, Institute of Acupuncture Research (WHO Collaborating Center for Traditional Medicine), Shanghai Medical College, Institutes of Brain Science, Fudan University, 138 Yi Xue Yuan Road, Shanghai 200032, China; ^2^Guiyang Medical University, 9 Beijing Road, Guiyang, Guizhou 550004, China; ^3^Department of Anatomy, Integrative Medicine College, Fujian University of Traditional Chinese Medicine, Qiu Yang Road, Minhou, Fuzhou, Fujian 350122, China

## Abstract

Cancer-induced bone pain (CIBP) is a severe type of chronic pain. It is imperative to explore safe and effective analgesic drugs for CIBP treatment. Baicalein (BE), isolated from the traditional Chinese herbal medicine* Scutellaria baicalensis* Georgi (or Huang Qin), has been demonstrated to have anti-inflammatory and neuroprotective effects. In this study, we examined the effect of BE on CIBP and the mechanism of this effect. Intrathecal and oral administration of BE at different doses could alleviate the mechanical allodynia in CIBP rats. Intrathecal 100 *μ*g BE could inhibit the production of IL-6 and TNF-*α* in the spinal cord of CIBP rats. Moreover, intrathecal 100 *μ*g BE could effectively inhibit the activation of p-p38 and p-JNK MAPK signals in CIBP rats. The analgesic effect of BE may be associated with the inhibition of the expression of the inflammatory cytokines IL-6 and TNF-*α* and through the activation of p-p38 and p-JNK MAPK signals in the spinal cord. These findings suggest that BE is a promising novel analgesic agent for CIBP.

## 1. Introduction

Cancer-induced bone pain (CIBP) results from multiple primary diseases, such as breast, lung, and prostate cancer, and severely impairs the quality of life of patients [1–3]. However, because of the complicated mechanism of CIBP and the lack of a proper animal model for studying CIBP, it remains a serious medical problem [4]. In the past two decades, several animal models have been established to help examine the mechanism of CIBP [5–7]. To date, results from our lab and others have demonstrated that CIBP is a complex pain state involving components of both neuropathic and inflammatory pain [5, 8, 9]. We observed significant levels of activated astrocytes and microglia cells and a subsequent robust increase in the expression of proinflammatory cytokines in the spinal cord of CIBP rats [10–12]. These cellular and molecular changes in the spinal cord are characteristic of neuroinflammation, which is critical for the development of CIBP.

Baicalein (BE) is a bioactive flavonoid derived from the root of* Scutellaria baicalensis* Georgi (or Huang Qin), which is widely used in Chinese herbal medicine [13, 14]. Evidence has shown that BE has many pharmacological effects, including antiallergic, antioxidant, antiapoptotic, antiviral, anti-inflammatory, antitumor, and neuroprotective effects and a modulatory effect on the immune system [15–19]. BE is also known as a nonselective 12/15-lipoxygenase (12/15-LOX) inhibitor that suppresses inflammation and proliferation and induces apoptosis in various cancers [20–22]. Whether BE can attenuate the neuroinflammation in the spinal cord that is characteristic of CIBP needs to be explored.

Therefore, the present study characterized the effect of BE on spinal neuroinflammation in CIBP rats. We hypothesized that the analgesic effect of BE is primarily due to the inhibition of the expression of proinflammatory mediators and the excessive activation of related signaling pathways.

## 2. Materials and Methods

### 2.1. Animals

Female Sprague-Dawley (SD) rats weighing 160–180 g were purchased from Shanghai Laboratory Animal Center, Chinese Academy Sciences. All animals were maintained in a climate-controlled room on a 12 h light-12 h dark cycle and were given unrestricted access to food and water. They were acclimatized to this for at least a week before the initiation of the experiments. All experimental procedures were approved by the Animal Care and Use Committee (ACUC) of Fudan University and were consistent with the NIH's Guide for the Care and Use of Laboratory Animals and the Ethical Issues of the IASP.

### 2.2. Surgical Procedures

Walker 256 rat mammary gland carcinoma cells were prepared as previously described [7, 23]. After the animals were anesthetized with sodium pentobarbital (i.p. 50 mg/kg), suspensions of 4 × 10^5^ tumor cells in 4 *μ*L 0.01 MPBS followed by 4 *μ*L of absorbable gelatin sponge dissolved in saline were slowly injected into the right tibias of each female SD rat using a 10 *μ*L microinjection syringe. The syringe was left in place for an additional minute after the injection to prevent the carcinoma cells from leaking out along the injection track. The sham group was treated in the same manner and was injected with 4 *μ*L PBS instead of 4 *μ*L of the tumor cell suspension.

### 2.3. Drug Administration

Different doses of BE (Sigma-Aldrich, St. Louis, MO, USA) were dissolved in dimethyl sulfoxide (DMSO) [24] (Sinopharm Chemical Reagent Co., Ltd., Shanghai, China) and administered in a volume of 20 *μ*L to rats via lumbar puncture, as previously reported [25]. Briefly, the rats were anesthetized with 2% isoflurane in air via a nose cone. For the experimental groups, a 29-gauge microinjection syringe filled with BE at different concentrations was inserted into the L5-6 interspace, and the control group was given the same volume of DMSO. We also observed the effect on CIBP rats of the oral administration of different doses of BE dissolved in 0.5% carboxymethylcellulose sodium (CMC) (Sinopharm Chemical Reagent Co., Ltd., Shanghai, China). The volume of oral BE was 2 mL.

### 2.4. Behavioral Test

Right hind paw withdrawal threshold (PWT) was determined in response to probing with a series of finely calibrated Von Frey Hairs (Stoelting, IL, USA) ranging from 1 to 26 g (1, 1.4, 2, 4, 6, 8, 10, 15, and 26 g). Hind PWT determination was performed as described previously [26]. Rats were placed in individual opaque boxes on a metal mesh platform. After the rats were acclimated to the test chambers for 30 min, the right hind PWT were determined by increasing and decreasing stimulus strength. Once the hind paw was withdrawn from a particular hair in three out of five consecutive applications, the rat was considered responsive to that hair, and the value of the filament in grams was considered to be the hind PWT for the mechanical stimuli. If hind paw withdrawal was not observed in three out of five consecutive applications, the next larger filaments in the series were tested consecutively until the rat withdrew the hind paw at least three times. The naive and sham-operated right hind paws were tested in the same manner. The investigator was blind to the groups being tested.

### 2.5. Real-Time PCR

The mRNA expression levels of proinflammatory cytokines were measured using real-time quantitative reverse transcription-polymerase chain reaction (RT-PCR). Total RNA was extracted from the L4–L6 spinal cord of the rats using TRIzol reagent (Invitrogen) according to the manufacturer's instructions. The mRNA levels of IL-1*β*, IL-6, TNF-*α*, and GAPDH were quantified by SYBR Green qRT-PCR detection (iCycler IQ real-time PCR detection system, Bio-Rad, Hercules City, California, USA), and each sample was run in duplicate. Total RNA samples of naive, cancer with DMSO, and cancer with BE rats were analyzed simultaneously by RT-PCR at the 2nd hour after drug injection. This time point was based on pain-related behavioral results that indicated the length of time after BE injection necessary to observe significant antihyperalgesic effects. The sequences of the PCR primers were as follows and as previously described by our laboratory: GAPDH, forward 5′-CCCTTCATTGACCTCAACTAC-3′ and reverse 5′-CTTCTCCATGGTGGTGAAGAC-3′; IL-1*β*, forward 5′-AGAGCTTCAGGAAGGCAG-3′ and reverse 5′-TGTTGTTCATCTCGAAGCCT-3′; IL-6, forward 5′-GACAAAGCCAGAGTCCTTCA-3′ and reverse 5′-ACTAGGTTTGCCGAGTAGAC-3′; and TNF-*α*, forward 5′-CGAGATGTGGAACTGGCAGA-3′ and reverse 5′-CTACGGGCTTGTCACTCGA-3′ [23, 27]. All primers were synthesized at Shanghai Sangon Biological Engineering Technology & Services Co., Ltd. Upon completion of the PCR, the relative amount of target message in each sample was estimated based on the threshold cycle number (Ct). The average Ct values for the indicated mRNAs were normalized to the average Ct values for the mRNA of the housekeeping gene GAPDH from the same cDNA preparations. These values were entered into the equation 2^−ΔΔCt^ to solve for the relative exponential PCR amplification of each gene for each animal. The results presented in this study are expressed as fold increases over control values.

### 2.6. Western Blotting

The rats (*n* = 4) were sacrificed on the 11th day after surgery. The L4-L5 spinal cords of the rats were analyzed in this study. Protein was extracted by homogenization in radioimmunoprecipitation (RIPA) lysis buffer (50 mM Tris (pH 7.4), 150 mM NaCl, 1% Triton X-100, 1% sodium deoxycholate, 0.1% sodium dodecyl sulfonate, sodium orthovanadate, sodium fluoride, ethylene diamine tetraacetic acid, and leupeptin) followed by centrifugation at 12000 g for 20 min [23, 28]. The protein concentrations of the supernatants were determined using the BCA Protein Assay Kit (Pierce, Rockford, USA), and 50 *μ*g of the proteins was loaded into each lane of a 10% SDS-PA gel for electrophoresis and then transferred onto polyvinylidene fluoride membranes. The membranes were blocked with 5% bovine serum albumin in TBS-T at 4°C for 2 h to prevent nonspecific binding. The membranes were then incubated with the primary antibodies rabbit anti-ERK1/2 (1 : 1000), rabbit antiphosphorylated ERK1/2 (1 : 2000), rabbit anti-p38 (1 : 1000), rabbit antiphosphorylated p38 (1 : 1000), rabbit anti-JNK (1 : 1000), rabbit antiphosphorylated JNK (1 : 1000), rabbit antiphosphorylated STAT3 antibody (1 : 1000), and mouse anti-STAT3 antibody (1 : 2000, Cell Signaling Technology, Beverly, MA, USA) overnight at 4°C. Then, the membranes were incubated with HRP-anti-rabbit/mouse (1 : 2000, Santa Cruz, CA, USA) antibody for 1 h at room temperature. The membranes were incubated with chemiluminescent reagents (ECL kit, Pierce, Rockford, Illinois, USA) for 1 min and then exposed using an ImageQuant LAS4000 mini image analyzer (GE Healthcare, Buckinghamshire, UK). The membranes were reblotted with GAPDH (1 : 10000, KangCheng, Shanghai, China). The intensities of the immunoreactive bands were quantified using ImageJ software.

### 2.7. Statistical Analysis

Data are presented as means ± standard error of the mean (SEM), and differences were analyzed for statistical significance by Student's *t*-test or one-way analysis of variance (ANOVA) followed by the Bonferroni posttest using the Statistical Package for the SPSS 17.0 statistical software (SPSS Inc., Chicago, IL). In all statistical analyses, *p* < 0.05 was considered to be the threshold for statistical significance.

## 3. Results

### 3.1. CIBP Caused by Inoculation with Walker 256 Cells

**Figure 1:**
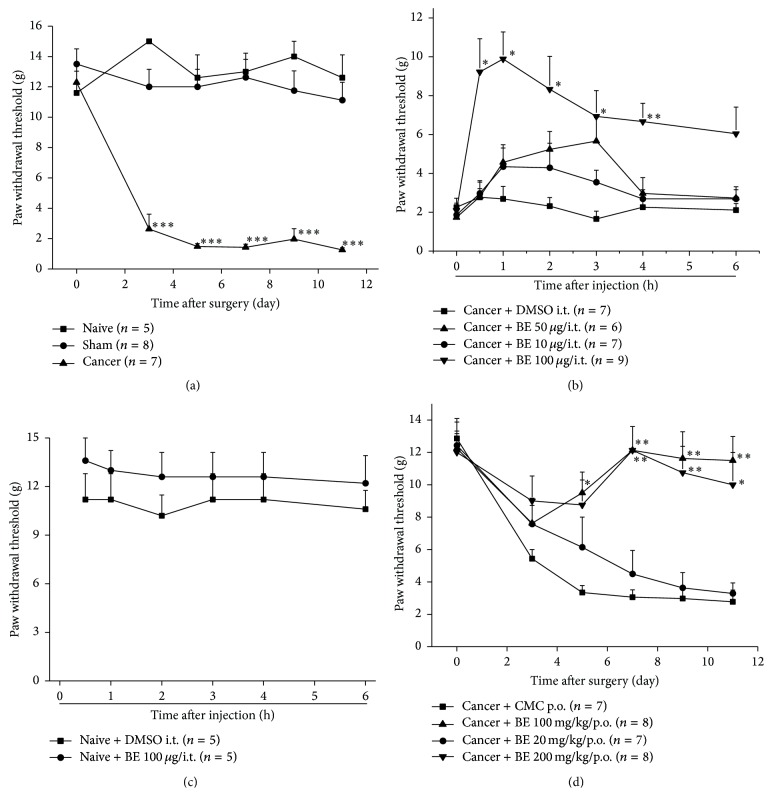
Intrathecal and oral administration of BE alleviates mechanical allodynia in CIBP. (a) Time course of the development of mechanical allodynia after cancer cell inoculation. Data are expressed as means ± SEM (^*∗∗∗*^ *p* < 0.001 versus the sham group). (b) Intrathecal BE alleviates mechanical allodynia in cancer rats. Data are expressed as means ± SEM (^*∗*^ *p* < 0.05, ^*∗∗*^ *p* < 0.01 versus cancer + DMSO group). (c) Intrathecal administration of BE has no influence on hind paw withdrawal threshold in naive rats. (d) Oral administration of different doses of BE increased hind paw withdrawal threshold in cancer rats. Data are expressed as means ± SEM (^*∗*^ *p* < 0.05 and ^*∗∗*^ *p* < 0.01 versus cancer + DMSO group).

Before inoculation with Walker 256 cells, there were no significant differences in the overall mean baseline hind PWT to Von Frey Hair stimulation among the naive, sham, and cancer groups. A significant decrease in the hind PWT of the cancer group was observed from the 3rd day after surgery compared to the naive group (Figure 1(a)). There were no differences in hind PWT between the naive and sham group during the observation window. These results suggest that mechanical allodynia developed in the inoculated hind paws of the rats in the cancer group.

### 3.2. BE Alleviated Mechanical Allodynia in CIBP Rats

The effect of BE was observed on the 11th day after surgery. Three doses of BE (10, 50, and 100 *μ*g) were given, and the 100 *μ*g dose of BE significantly alleviated the mechanical allodynia observed in the cancer group from 0.5 h to more than 4 hours after intrathecal injection (Figure 1(b)). Intrathecal administration of BE had no effect on the hind PWT of naive rats (Figure 1(c)). We also observed an analgesic effect of BE by oral administration. Three doses of BE (20, 100, and 200 mg/kg) were orally administered from the 1st day to the 10th day after surgery. We observed an analgesic effect of the 100 mg/kg and 200 mg/kg doses of orally administered BE; however, the 20 mg/kg dose of BE had no effect (Figure 1(d)).

### 3.3. Intrathecal BE Reduced the Expression of Proinflammatory Cytokines and Inhibited the Activation of p38 and JNK MAPK Signals in the Spinal Cord of CIBP Rats

**Figure 2:**
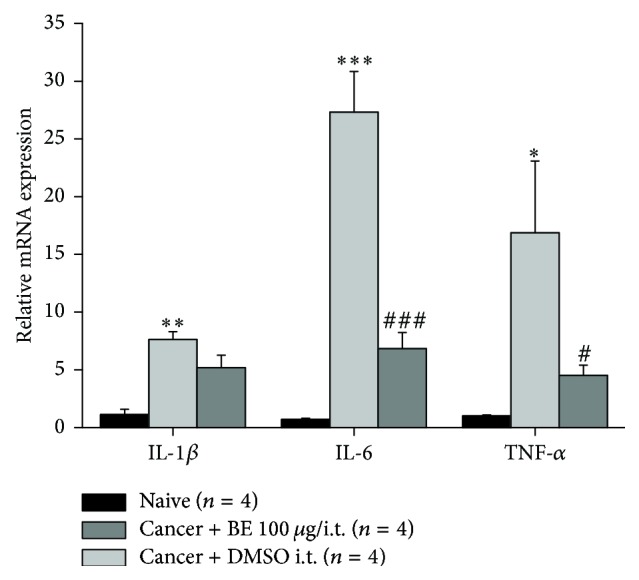
Intrathecal BE inhibited the expression of proinflammatory factors (IL-6 and TNF-*α*) in spinal cord of CIBP rats. Intrathecal 100 *μ*g BE markedly downregulated the expression of IL-6 and TNF-*α* mRNA. Data are presented as means ± SEM (^*∗*^ *p* < 0.05, ^*∗∗*^ *p* < 0.01, and ^*∗∗∗*^ *p* < 0.001 versus naïve group; ^#^ *p* < 0.05 and ^###^ *p* < 0.001 versus cancer + DMSO i.t. group).

To explore the mechanism of the analgesic effect of BE, we examined the mRNA expression of proinflammatory cytokines in the spinal cord of CIBP rats on the 11th day after surgery by RT-PCR analysis. Consistent with previous reports, significant upregulations of IL-1*β*, IL-6, and TNF-*α* mRNAs in the cancer group compared to the naive group were observed (Figure 2) [10, 29]. Intrathecal administration of 100 *μ*g BE reversed the upregulation of IL-6 and TNF-*α* observed in the cancer group (Figure 2) at the 2nd h after injection. However, the upregulation of IL-1*β* in the cancer group was not inhibited by BE (Figure 2).

**Figure 3:**
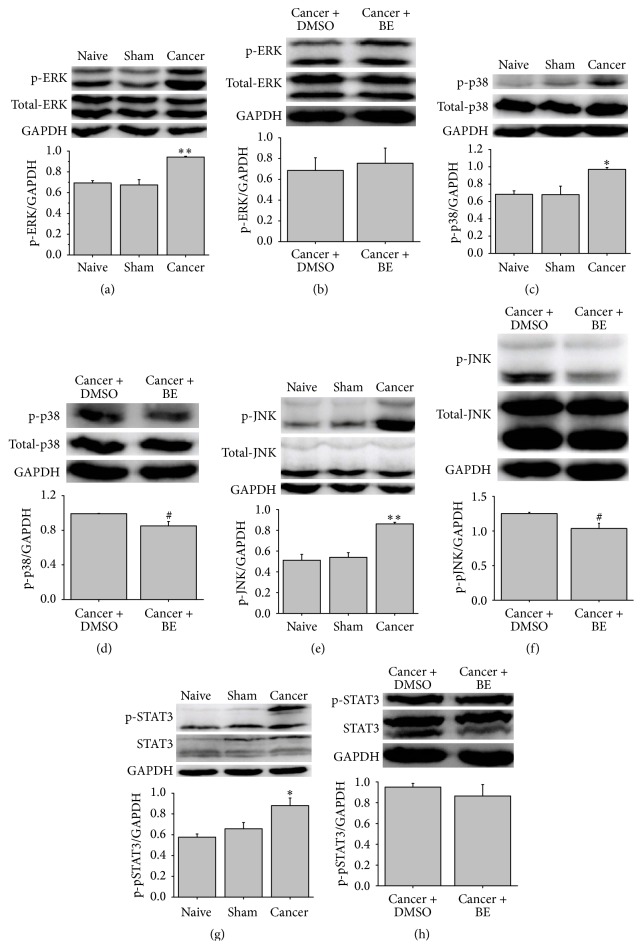
Intrathecal BE inhibits the expression of p-p38 and p-JNK in spinal cord of CIBP rats. ((a), (b)) Western blot and the quantitative data of p-ERK protein in spinal cord of rats. Protein level of p-ERK significantly increased in CIBP rats compared to naïve rats. However, intrathecal BE (100 *μ*g) had no influence on the expression of p-ERK. ((c), (d)) Western blot and quantitative data of p-p38 protein in spinal cord of rats. Intrathecal BE (100 *μ*g) reduced the expression of p-p38 protein in CIBP. ((e), (f)) Western blot and quantitative data of p-JNK protein in spinal cord of rats. Intrathecal BE (100 *μ*g) reduced the expression of p-JNK in CIBP. ((g), (h)) Western blot and quantitative data of p-STAT3 protein in spinal cord of rats. Intrathecal BE (100 *μ*g) had no influence on the expression of p-STAT3 in CIBP. Data are presented as means ± SEM (^*∗*^ *p* < 0.05, ^*∗∗*^ *p* < 0.01 versus naïve group; ^#^ *p* < 0.05 versus cancer + DMSO i.t. group).

Because MAPK signals are closely linked to CIBP, previous works have demonstrated that the inhibition of the activation of p-ERK, p-p38, and p-JNK significantly alleviates mechanical allodynia in CIBP rats [28, 30–32]. At 2 h after the intrathecal administration of BE on the 11th day after surgery, we examined the expression of MAPK signals by Western blot. Consistent with previous reports, the protein levels of p-ERK, p-p38, and p-JNK in the cancer group were significantly elevated compared to those of the naive group (Figures 3(a), 3(c), and 3(e)). Intrathecal BE inhibited the activation of p-p38 and p-JNK (Figures 3(d) and 3(f)). However, p-ERK protein expression was not influenced by BE (Figure 3(b)).

The results of the real-time PCR analysis indicated that the expression of the proinflammatory cytokine IL-6 was significantly increased in CIBP rats compared to the naive group. Intrathecal administration of BE reversed this increase in IL-6 expression (Figure 2). It is well known that the IL-6 signal acts through Janus kinases/signal transducers and activators of transcription (JAKs/STATs), in particular STAT3 [33, 34]. The JAK/STAT3 signal plays an important role in tumor cell proliferation, survival, and invasion and participates in the development of inflammation and neuropathic pain [34–36]. We examined the expression of STAT3 in CIBP rats. A significant activation of p-STAT3 expression was observed in the cancer group compared to the naive group; however, intrathecal administration of BE did not inhibit the activation of p-STAT3 in the cancer group (Figures 3(g) and 3(h)).

## 4. Discussion

Here, we showed for the first time that BE produces an analgesic effect in CIBP rats. The mechanism of BE's analgesic effect involved the attenuation of the expression of the proinflammatory cytokines IL-6 and TNF-*α* and the inhibition of the activation of p-p38 and p-JNK MAPK signals.

CIBP is the most severe type of chronic pain and has received great attention in clinical research. Analgesic drugs used in clinical settings, such as nonsteroidal anti-inflammatory drugs (NSAIDs) and morphine, are limited because of their side effects, such as gastrointestinal reactions, addiction, and tolerance [8]. Thus, it is imperative to find safe and effective analgesic drugs for CIBP. Previous reports have demonstrated that the neuroinflammation that occurs in the spinal cord of CIBP rats plays an important role in CIBP [9, 11, 37]. Thus, we hypothesized that inhibiting neuroinflammation may alleviate mechanical allodynia in CIBP rats.

BE is one of the most important flavonoid compounds that have been identified to date, and it was purified from Huang Qin. BE has been reported to possess several pharmacological activities and has been used in the treatment of a variety of different diseases, such as osteoarthritis, bronchitis, pulmonary fibrosis, dengue virus, diabetes, hepatoma, breast cancer, Parkinson disease, and Alzheimer disease [13, 16–18, 38]. Huang Qin has been reported to be one of the most important herbs used for medical purposes, and PHY906, a four-herb Chinese medicine formula (consists of four herbs:* Glycyrrhiza uralensis* Fisch.,* Paeonia lactiflora* Pall.,* Scutellaria baicalensis* Georgi, and* Ziziphus jujuba* Mill.), can greatly reduce chemotherapy-induced gastrointestinal toxicity and repress the transcription of nuclear factor *κ*B and immune-suppression in tumors [39]. Moreover, the results of a study by Gregus et al. demonstrated that intrathecal delivery of 10 *μ*g BE prevented carrageenan-evoked hyperalgesia for more than 4 h [40]. Thus, the present study assessed the analgesic effect of BE in CIBP rats. The behavioral tests performed in this study demonstrated that the intrathecal injection of 100 *μ*g BE significantly alleviated mechanical allodynia for more than 4 h in CIBP rats. BE doses of 10 *μ*g and 50 *μ*g only showed a little upregulated hind PWT after administration. These results are different from those of Gregus et al., possibly because of the different pain models used in these two experiments. It has also been demonstrated that the mechanism of CIBP is different from that of inflammatory pain [9, 41].

We also observed the effect of different doses of BE (150 and 300 mg/kg) administered intraperitoneally to CIBP rats; these doses were chosen based on a report of the effect of BE in a cerebral ischemia/reperfusion model [42]. Only the 300 mg/kg dose of BE alleviated the mechanical allodynia in the CIBP rats, and this was only observed at the 2nd h after its administration (data not showed). The mechanism of CIBP may be different from that of stroke, and the CIBP rats displayed persistent malignant symptoms for a long period of time. A single peritoneal injection of BE may be insufficient and less effective than multiple injections for the alleviation of mechanical allodynia in CIBP rats. It has been reported that daily administration of BE at 250 mg/kg through gastric gavages reduced proliferation and induced apoptosis in rat hepatocellular carcinoma cells, and, in a model of bleomycin-induced pulmonary fibrosis, oral administration of 50 and 100 mg/kg BE daily from days 1 to 28 after the induction of fibrosis significantly attenuated the fibrosis [43, 44]. We chose the BE doses of 20, 100, and 200 mg/kg for oral administration for 10 days from the 1st day after surgery, and the 100 and 200 mg/kg doses of BE alleviated the mechanical allodynia in CIBP rats.

The proinflammatory mediators IL-1*β*, IL-6, and TNF-*α* were upregulated in the spinal cord of CIBP rats. BE inhibited the mRNA expressions of IL-6 and TNF-*α* but not of IL-1*β*. This may indicate that the anti-inflammatory effect of BE in CIBP does not involve the IL-1*β* signaling pathway.

The MAPK signaling pathway belongs to the receptor tyrosine kinase family and plays a critical role in tumorigenesis and chronic pain. The MAPK signaling molecules p-ERK, p-p38, and p-JNK have been shown to be significantly activated in CIBP rats. Intrathecal administration of any of the selective inhibitors U0126, SB203580, and SP600125 alleviated mechanical allodynia in CIBP rats [28, 31, 32]. BE has an antihyperalgesic effect in CIBP rats, so we examined the influence of BE on the activation of MAPK signals in CIBP rats. The results demonstrated that intrathecal BE significantly reduced the activation of p-p38 and p-JNK. Previous studies have demonstrated that the activation of the p38 MAPK signal increases the transcription of nociceptive peptides, such as SP (substance protein) and CGRP (Calcitonin gene-related peptide). Moreover, the p38 MAPK signal has been shown to mediate injury-induced increases in the production of TRPV1 and TRPA1 in models of neuropathic pain and inflammatory pain [31, 45, 46]. The activation of the p-JNK MAPK signal and in particular the MAPK signaling molecule JNK1 plays an important role in the maintenance of persistent neuropathic pain, inflammatory pain, and CIBP [47, 48]. Thus, the analgesic effect of BE in CIBP rats may be, at least partially, due to the inhibition of the activation of p-p38 and p-JNK MAPK signals.

The JAKs/STATs pathway is involved in the regulation of cellular responses involved in the activation and proliferation of bone marrow-derived cells [34]. However, excessive activation of JAKs/STATs signals, in particular JAK/STAT3, critically contributes to tumorigenesis [34, 49]. STAT3 is known to play an essential role in IL-6 signaling in the central nervous system [33, 35]. Previous reports have demonstrated that inhibiting the significant activation of the JAK/STAT3 pathway helps alleviate neuropathic pain [35, 36]. Thus, we examined the expression of p-STAT3 after intrathecal injection of BE. However, the significant activation of p-STAT3 was not influenced by BE in CIBP rats. These results indicated that the analgesic effect of BE does not involve the JAK/STAT3 pathway and that the mechanism of neuropathic pain is different from that of CIBP.

## 5. Conclusions

These results demonstrate that BE effectively alleviates mechanical allodynia in CIBP rats. The effects of BE are associated with reducing the expression of the proinflammatory cytokines IL-6 and TNF-*α* and inhibiting the activation of the p-p38 and p-JNK MAPK signal pathways. These results suggest that BE plays an analgesic effect in CIBP rats, at least partially, by inhibiting neuroinflammation in the spinal cord.
